# The clinical evaluation of *Basti* along with *Rasayana* on symptoms of post-COVID-19 syndrome: an open-labeled proof of concept pragmatic study—a study protocol

**DOI:** 10.1186/s40814-023-01322-1

**Published:** 2023-06-03

**Authors:** Amit Nakanekar, Payal Rathod

**Affiliations:** Government Ayurved College, Nagpur, Maharashtra India

**Keywords:** Ayurved, *Basti*, COVID-19, Post-COVID-19, Pragmatic, *Rasayana*

## Abstract

**Background:**

Post-COVID-19 syndrome is a result of triggering various immune pathways and metabolic disturbances. *Basti* is an important per rectal Ayurveda-based treatment having multi-targeted actions. *Basti* and *Rasayana* treatment modulate immune responses by regulating pro-inflammatory cytokines, immune globulins, and functional properties of T cell. We propose to study the clinical evaluation of *Basti* along with *Rasayana* (rejuvenation therapy) on symptoms of post-COVID 19 syndrome.

**Methods and analysis:**

We designed a prospective, open-labeled proof of concept pragmatic study. The study duration is 18 months, and the intervention period are 35 days from the day of enrollment of the patients. The patients will be treated on the basis of Ayurvedic classification of *Santarpanottha* (over nutrition) symptoms and *Apatarpanottha (lack of nutrition)* symptoms. The *Santarpanottha* group will be treated within 3–5 days of oral *Guggulu Tiktak Kashayam* followed by 8 days of *Yog Basti* treatment and then 21 days of *Rasayana* therapy with *Brahma Rasayan*. The *Apatarpanottha* group will be treated within 3–5 days of oral *Laghumalini Vasant*, followed by 8 days of *Yog Basti* treatment and then 21 days of *Kalyanak Ghrit.*

The outcome measures of this study will be to evaluate the changes in fatigue severity scale, MMRC dyspnea chest pain scale, pain score assessed by VAS scale, smell and taste scale, WOMAC scale, Hamilton depression scale, Hamilton anxiety scale, Insomnia Severity Index, change in Cough Severity Index, facial aging scale, dizziness scale, Pittsburgh Sleep Severity Quality Index, functional status scale, and heart palpitation scale.

All adverse events will be monitored at each time throughout the study visit time. A total of 24 participants will be recruited to demonstrate with 95% confidence interval and 80% power.

**Discussion:**

Ayurveda treats *Santarpanottha* (originated from over nutrition) symptoms and *Apatarpanottha* (symptoms originated from undernutrition) symptoms differently; hence, inspite of the same disease or symptom management, changes depend upon the type of the origin. This pragmatic clinical study is developed on the fundamental grounds of Ayurveda.

**Ethics and dissemination:**

Ethics approval was obtained through the Institutional Ethics Committees of Government Ayurved College and Hospital on 23 July 2021.

**Trial registration:**

The trial is prospectively registered with the Clinical Trial Registry of India on 17 August 2021 [CTRI/2021/08/035732] after the Institutional Ethics Committee approval [GACN/PGS/Synopsis/800/2021 Date 23/7/2021].

**Supplementary Information:**

The online version contains supplementary material available at 10.1186/s40814-023-01322-1.

## Introduction

About 117 million people have been diagnosed with COVID-19 globally, with more than 2.6 million deaths till March 2021. It is caused by the novel severe acute respiratory syndrome coronavirus 2, i.e., SARS-CoV-2, in which heterogeneous virus manifests itself with a wide spectrum of symptoms, from asymptomatic to a life-threatening and fatal disease [1]. After COVID-19, some patients experience symptoms which are termed as a post-COVID-19 syndrome.

Post-COVID-19 syndrome can be seen in all those who had COVID-19, even if the illness was mild or with no symptoms. The commonly reported symptoms for post-COVID-19 syndrome are chronic fatigue, dyspnea, shortness of breath, chest pains, headache, loss of smell, loss of taste, and muscle and joint pain, followed by insomnia, depression, anxiety, itchy body, tachycardia, heart palpitations, tachycardia, anorexia, tingling fingertips, and brain fog [1]. A cytokine profile that is very specific is associated with several factors in the severe stage of this disease. It includes the induction of interferon production, interleukin (ILs), cytokine secretion, stimulation of granulocyte activation, and production of tumor necrosis factor (TNF). This causes intravascular hyperinflammation with changes in angiogenesis and coagulation in addition to the understanding of the immune response to COVID-19. Post-COVID-19 symptoms and their association with autoimmune diseases suggest that SARS-CoV-2 may trigger various diseases associated to the immune system and metabolism.

Viral infection leads to an aggressive immunological reaction on cardiopulmonary system, nervous system, hematological system, gastrointestinal tract, liver and kidney, and various other systems of the human body. SARS-CoV-2 infection can lead to destabilization of the liver that signals maximum alertness to whole organism and generates an increase in production of plasma biochemical markers of the tissue aggression in response to tissue-damaging agents [2]. Various complex molecular processes are responsible for post-COVID-19 syndrome, and targeting all of them at a time is a difficult task.

The gastro-intestinal tract is the largest immunological organ in the body, and its resident microbiota are known to modulate host responses. In post-COVID-19 syndrome, gut microbiota composition is disturbed and it is also associated with disease severity and plasma concentrations of several cytokines and inflammatory markers. The patients with COVID-19 report persistent symptoms after recovery and subsequently develop dysbiotic gut microbiota. There are various immune-related health problems post-COVID-19 [3].

COVID-19 is correlated with *Abhishangaj Jvara (fever* arising due external cause*)* in *Ayurveda* [4]*.* Some patients who suffered from COVID-19 followed *Langhana* (fasting) while some have not followed [5]. Therefore, currently observed signs and symptoms of post-COVID-19 syndrome can be categorized into two groups, i.e., *Santarpanottha* (excessive nutrition) and *Apatarpanottha* (poor nutrition) symptoms as per correlations with *Astang Hrdayam Sutrasthan* 14 [6]. COVID-19 epidemic can be termed as “*Janapadodhvans Vyadi* (epidemic)”*.* Charka described this concept of epidemic, which have a causing factor like polluted *Vayu* (air)*, Jala* (water)*, Desh* (country), and *Kaal* (time). *Panchakarma* (five cleansing process) and *Rasayana* (rejuvenation) therapy are two important treatments that are advised by Charka for various *Janapadodhvans Vyadi* [7, 8].

It is evident that COVID-19 leads to debilitation, and condition is more likely *Apatarpanothha*.

Recent articles have shown that post-COVID metabolic dysfunction can lead to insulin résistance and obesity. Recent researches have that there are metabolic clues that can lead to bidirectional pathologies.

Obesity, insulin resistance, development of obese type 2 DM may fall under *Santarpanottha*.

*Basti* (medicated enema) is one of the important *Panchkarma* used for many disorders; it has multitargeted action, hence many times, it forms an important part of the treatment, termed as “*Ardha Chikitsa”* [9]. Based on type of medicines used, *Basti* (medicated enema) can treat both *Santarpanottha* (excessive nutrition) as well as *Apatarpanottha* (poor nutrition) diseases. *Basti* (medicated enema) removes toxins, and it rejuvenates the body also. *Basti* is mentioned as a *Ardha Chikitsa* in classical ayurvedic texts, hence for broader purpose, we choose *Basti* [10]. *Vata* is an important Dosha that can show maximum number of *Nanatmaj Vyadhi* (various diseases), and it is also responsible for the movement of other *Doshas* [11]*.* *Basti* is an important treatment for *Vata dosha* [12]*.* Thatte et al. have shown that *Basti* modulates immune responses by regulating pro-inflammatory cytokines, immune globulins, and functional properties of T cell [13]. Rastogi et al. showed that *Rasayana* act as rejuvenation therapy, anti-stress, antioxidant, anti-inflammatory, anti-microbial, vaccine adjuvant, and confer immunity against various diseases [14]. We have decided to evaluate the efficacy of *Basti* and *Rasayana* on post-COVID-19 syndrome.

## Objectives

1. To evaluate the efficacy of Guggulutiktak Kashayam followed by *Yog Basti* (*Triphala Decoction Niruh Basti, medicated oil Anuvasan Basti*) followed by *Brahma Rasayana* on symptoms of *Santarpanottha* post-COVID-19 syndrome in the period of 35 days.
2. To evaluate the efficacy of tablet Laghumalini Vasant followed by *Yog Basti* (*Brihatpanchmulsidhha Kshirbasti [medicated milk enema], Kshirbala tail [medicated oil* processed in milk*] Anuvasan Basti*) followed by *Kalyanak Ghrit* (medicated cow ghee) on symptoms *Apatarpanottha* post-COVID-19 syndrome in period of 35 days.

## Methods and analysis

### Patient and public involvement

This study protocol is developed to evaluate the effect of *Basti* and *Rasayana* therapy to modulate immune responses in post-COVID-19 syndrome. Patients were not involved in the design, recruitment, or conduct of the study. The details of procedures with times are explained in Table 1.

**Table 1:** Summary of activities

	Enrollment	Allocation	Post allocation			Completion
Timepoint	Screening Day 0	Day 1	Days 3 to 5	Days 5 to 13	Days 14 to 34	Day 35
**Enrollment**						
Eligibility criteria	×					
Informed consent	×					
Allocation		×				
**Interventions**						
Daily doses of drugs and *Basti* therapy						
**Assessments**						
Concomitant illness	×	×	×	×	×	×
Complete physical examination	×	×	×	×	×	×
Signs and symptoms assessment	×				×	×
Assessment of vitals	×	×	×	×	×	×
Ayurvedic evaluation	×	×	×	×	×	×
*Basti* charting				×		
Clinical scales assessment	×				×	×
Study drug dispensation		×	×	×	×	
compliance			×	×	×	×
Fatigue severity scale	×				×	×
MMRC dyspnea scale	×				×	×
Chest pain scale	×				×	×
VAS	×				×	×
Smell and taste scale	×				×	×
WOMAC Scale	×				×	×
Hamilton depression scale	×				×	×
Hamilton anxiety scale	×				×	×
Insomnia Severity Index	×				×	×
Cough Severity Index	×				×	×
Facial aging scale	×				×	×
Dizziness scale	×				×	×
Pittsburgh severity quality index	×				×	×
Functional status scale	×				×	×
Heart palpitation scale	×				×	×
Completion and outcome						×

### Study design

**Fig. 1:**
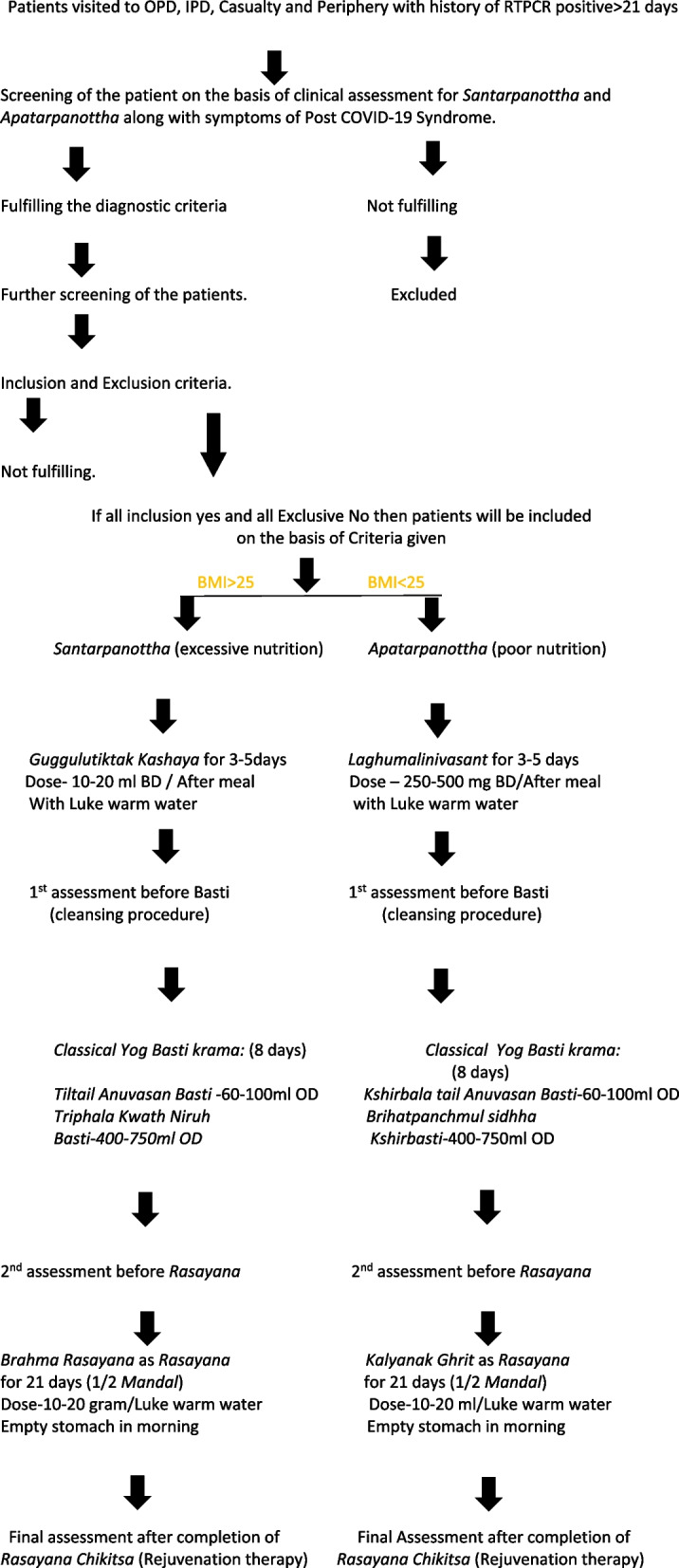
Flow chart of study

This is a prospective, open-labeled proof of concept pragmatic study. The study duration is 18 months, and the intervention period is 35 days from the day of enrollment of the patients. The trial will be conducted at OPD, IPD of Kayachikitsa, Casualty and Periphery of Government Ayurved College and Hospital, Nagpur. Clinical assessment and Ayurveda-based clinical assessment will also be carried out. The detailed flow chart of process is shown in Fig. 1.

### Recruitment, screening, and consent

The participant of the age group 18–70 years will be selected for study. The study will include up to completion of 24 participants.

The investigator number 2 (PR) will be responsible for obtaining written informed consent from each participant prior to screening as per the national ethics guidelines [15]. An information sheet or case record format in a language best understood by the participant, giving details of the study, will be provided to the participant. Enough time will be given to the participant to make a voluntary decision to participate. The participant will be encouraged to ask questions and clear all doubts in a face-to-face interview with the investigator. After that, the participant will be asked to sign the consent form for the study research. The participants will be screened from the patients with post-COVID-19 syndrome complaints comprehensively by the study physicians for eligibility and to exclude any contraindication to participation in the study. If found eligible, the participants will be enrolled. The participants will be followed up at predetermined time endpoints (five visits including screening and completion visits) as per protocol till study completion (35th day). A detailed procedure is summarized in Fig. 1 and Table 1. Data will be recorded in case record form and will be then entered in an Excel sheet.

### Eligibility

#### Inclusion criteria

The patients of either sex aged between 18 and 70 years are the ones included in the study, irrespective of caste, religion, socioeconomic, and educational status as long as they are willing to participate and have a history of positive RTPCR report under duration of > 21 days to 1 year. Those patients having > 20% (≥ 4) of symptoms will be included for the study and patients of post-COVID 19 syndrome who are willing to participate in the trial and are ready to give consent.

#### Exclusion criteria

Patients with serious life-threatening diseases and critically ill patients of post-COVID-19 syndrome will be excluded. Pregnant females and lactating mothers will not be considered for the study. Patients who will be unfit for *Basti* will be excluded from the study. Patient who has known hypersensitivity reactions to medicines mentioned in the study will be excluded. Any other patient whom investigator feels not to be included will be excluded from the study.

#### Withdrawal criteria

During the course of this treatment, if any serious condition or emergency condition develops, the required treatment will be given immediately to patients and such subject may be withdrawn from the study. Noncompliance patients will be withdrawn from the study. Patients selected for this study who are not following instructions properly will be considered as dropout.

### Monitoring

At zero day, the participants will be physically checked by the study physicians at all visits till the completion of the study. At all time points, assessment of participants will also include in-depth inquiry of possible symptoms of COVID-19 in the family members and close associates. The daily response of the participant will be seen by the physician and study co-ordinators. In addition, a telephonic contact will be made with the participant by the coordinator once in every week till the completion of the study to find out about any matter concerning health, adverse event, or the study. The schedule of enrollment, interventions, and assessments is provided in Table 1.

### Compliance

The participants will be daily monitored for symptoms of post-COVID-19 syndrome and related to *Basti* therapy-related side effects. Telephonic follow up will be done during the 2th, 3th, and 4th visits throughout the study. The study coordinator will contact the participants to monitor symptoms and ensure compliance. Medication logs will be maintained for intervention drug consumption. The drug compliance will be assessed at each follow-up visit by the patients and counting the number of empty containers returned and assessing the quantity of medicines consumed by the patient. Also, drug compliance report form will be provided to the study patients and filled by the patients and submitted to the investigators during the follow-up visit.

### Trial intervention

An intervention will be decided on the basis of *Santarpanottha* symptoms or *Apatarpanottha* symptoms.

#### Intervention arm (Group 1)

The *Santarpanottha* group will be treated in three stages: *Pachan, Shodhan,* and *Rasayan* in a sequence as follows:

- *Pachan* *(*conversion of toxins in removable form)—Ayurveda formulation *Guggulutiktak Kashaya* 10–30 ml BD orally after meals with warm water and *Gomutra arak* (cow urine distillate) for 3 to 5 days will be given. *Guggulutiktak Kashaya* is a classical Ayurvedic decoction used to treat diseases arising from over nutrition.
- *Shodhan* (removal of toxins)—Classical *Ayurveda Yog Basti* treatment includes 8 days of per rectal medications as per classical Ayurved methods, i.e., *Anuvasan Basti* and *Niruh Basti* will be administered on alternate days. Processed sesame oil will be used for *Anuvasan Basti* 60–100 ml per rectally after meal OD given for alternate 3 days, and *Triphala* decoction mixed with processed sesame oil and cow urine distillate will be used for classical *Niruh Basti*. The dose of *Niruha Basti* will vary between 400 and 850 ml empty stomach OD given for alternate 3 days. The last two *Basti* will be *Anuvasan* that is processed sesame oil after meal. One will be given either *Anuvasan* or *Niruha* on alternate days from the 1st to 6th day. In the last 2 days, i.e., the 7th and 8th day, *Anuvasan* will be given.
- *Rasayan* (rejuvenation)—After completion of classical *Yog Basti* treatment, *Rasayana* treatment will be given for 21 days. *Brahma Rasayana* will be used as a *Rasayana* in the dose of 10–30 g OD orally on an empty stomach in the morning with warm water for 21 days.

The exact dose for each patient will be finalized on the basis of Ayurveda-based parameters like *Koshtha*, *Agni*, and *Basti Nirgaman*.

#### Intervention arm (Group 2)

The *Apatarpanottha* group will also be treated in three stages: *Pachan, Shodhan, and Rasay*an in sequence as follows:

- ***Pachan*** (conversion of toxins in removable form)—Ayurveda formulation Tablet Laghumalini *Vasant* 250–500 mg BD orally after meals with water for 3 to 5 days.
- ***Shodhan*** (removal of toxins)—*Yog Basti* treatment includes 8 days of per rectal medications as per classical Ayurved methods, i.e., *Anuvasan Basti* and *Niruh Basti* will be administered on alternate days. *Kshirbala oil* will be used for *Anuvasan Basti* 60–100 ml per rectal after meal OD for 5 days. Milk processed in *Brihat Panchmula herbs* will be used for *Niruha* per rectal in dose 400–850 ml on an empty stomach OD given for alternate 3 days. One will be given either *Anuvasan* or *Niruha* on alternate days from the 1st to 6th day. The last 2 days, i.e., the 7th and 8th day, *Anuvasan* will be given.
- ***Rasayan*** (rejuvenation)—After completion of classical *Yog Basti* treatment, *Rasayana* treatment will be given for 21 days. *Kalyanak Ghrit* will be used 15–30 ml OD orally on an empty stomach in the morning with warm water for 21 days.

The exact dose for each patient in both groups will be finalized on the basis of Ayurveda-based parameters like *Koshtha, Agni*, and *Basti Nirgaman* (evacuation).

### Clinical outcomes

#### Primary outcome

1. To evaluate the efficacy of Guggulutiktak Kashayam followed by *Yog Basti* (*Triphala Kwath Niruh Basti, Tiltail Anuvasan Basti*) followed by *Brahma Rasayana* on symptoms of *Santarpanottha* post-COVID-19 syndrome in the period of 35 days.
2. To evaluate the efficacy of Laghumalini Vasant followed by *Yog Basti* (*Brihatpanchmulsidhha Kshirbasti, Kshirbala tail Anuvasan Basti*) followed by *Kalyanak Ghrit* on symptoms *Apatarpanottha* post-COVID-19 syndrome in the period of 35 days.

#### Secondary outcomes

1. Change in fatigue score assessed by fatigue severity scale
2. dyspnea score assessed by MMRC dyspnea scale
3. chest pain assessed by chest pain scale
4. pain score assessed by VAS scale
5. Change in score assessed by smell and taste scale
6. Change in score assessed by WOMAC scale
7. Depression score assessed by Hamilton depression scale
8. Anxiety score assessed by Hamilton anxiety scale
9. Change in score assessed by Insomnia Severity Index
10. Change in Cough Severity Index
11. Change in score facial aging scale
12. Change in score assessed by dizziness scale
13. Change in sleep quality assessed by Pittsburgh Severity Quality Index
14. Change in the quality of life assessed by functional status scale
15. Change in score assessed by heart palpitation scale

### Adverse events

All adverse events (AEs) during the therapy will be recorded and monitored as per ICH-GCP (2016) [15] and ICMR guidelines [16]. The safety of the participants would also be assessed in case of all withdrawals. All adverse events will be recorded and followed till resolution. All adverse events will be classified by the study physician and recorded in the AE form. The Data Safety Monitoring Board (DSMB) will be formed to monitor the safety of the participants and assess the safety data. Clinical assessments will be carried out as per Table 1.

### Concomitant medication

For the patients registered under the trial study, investigators will be instructed to avoid the use of other drugs on their own view and consult the investigators for any illness, symptom, and complaint or if they feel anything unusual. The investigator will record any medication(s) he/she may have prescribed for this any emergency medical condition, and the use of any concomitant medication will be prescribed only by the investigator mentioned in its case record form.

### Statistical analysis

#### Sample size and power

The sample size for the study is calculated by using the formula with assumption which is made after the pilot study on 4 patients. The assumption made is that our treatment will reduce total symptoms by at least 40% with 95% confidence interval (*α* = 0.05) and 80% power of the study and expecting a dropout rate of 20%. Hence, a total of 24 patients will be recruited.

#### Analysis

Results and observation seen in the research will be analyzed by proper statistical methods. The data collected in the CRF will be computerized in an Excel file for a separate group. Wilcoxon signed-rank test, Mann–Whitney, chi-square, and non-parametric ANOVA will be carried out to evaluate the effect on subjective criteria. Paired *t* test, unpaired *t* test, and ANOVA will be carried for normally distributed parametric/objective criteria**.**

## Discussion

In COVID-19 and post-COVID-19, a cytokine profile that is very specific is associated with several factors in the severe stage of this disease. It includes induction of interferon production, interleukin (ILs), cytokine secretion, the stimulation of granulocyte activation, and the production of tumor necrosis factor (TNF). The patients with COVID-19 report persistent symptoms after recovery and subsequently develop dysbiotic gut microbiota. Ayurveda states that all the disease originated from gut and disturbed gut microbiota can lead to various systemic diseases and immunological diseases, and *Basti* therapy (medicated enema) will be given on gut site, and they can detoxify the system and improve the health of gut.

Our intervention includes *Pachan* treatment to improve gut and digestive system for cleansing procedure.

- *Guggulutiktak Kashaya—*A classical *Ayurvedic* decoction that contains herbs like *Nimba (Azadirachta indica), Amruta (Tinospora Cordifolia), Vasa (Adhatoda Vasica), Kantakari (Solanum Virginianum), Goghrit (Clarified butter),* and *Guggul (Commiphora Wightii). Panchtikt* has *Vishamjvar nashan* property [17].
- This can also be used in various post-COVID conditions related to obesity, insulin resistance, chronic inflammation in joints, etc.
- *Triphala Kwath—*It is a decoction of *Haritaki (Terminalia Chebula), Bibhitaki (Terminalia Bellirica),* and *Amalaki (Phyllanthus Embilica L).* It has *Kaphapittaghna* and *Vishamjvar Nashan* property [18]. Triphala also has antiobesity and neuroprotective properties. It also acts on functional gut disorders that can be seen in post-COVID-19 [19, 20]. Various studies have shown that Triphala can also target different target proteins involved in pathophysiology of post-COVID syndrome [21].
- *Laghumalini Vasant*—It contain *Kharpar (zinc ore), Marich (Piper Nigrum), Navneet* (fresh butter), and *Niburasa (Citrus Limon)*. It has property to treat *Jvara (fever), Vishamjvar,* and act originated after as on multiple diseases a consequence of fever [22].
- *Brihatpanchmulsidhha Siddha Kshirbasti*—It contains *milk* processed in various herbs *Bilva (Aegle Marmelos), Agnimantha (Premna Serratifolia), Shounak (Oroxylum Indicum), Patala (Sterospermum Suaveolens)*, and *Gambhari (Gmelina Arborea). It* has *Brihan (excessive nutrition)* effect and *Tikta* dominant *Rasa* [23]. *Siddha* with *Dugadhha (cow milk)* increases potency of drug and treat diseases of chronic origin [24]. It can be useful in post-COVID weakness, giving strength to the lungs and decreases chronic inflammation.
- *Brahma Rasayana—*It is a classical Ayurvedic formulation which has important ingredients of *Haritaki (terminalia chebula)* and *Amalaki (Phyllanthus embilica L)* which has a property to treat various diseases like cough and weaknesses. It increases *Medha, Smiriti*, and *Bala* and acts as rejuvenation. This can also improve neurocognitive functions [25].
- *Kalyanak Ghrit*—It is a medicated ghee described in Charak Samhita. All ingredients of *Kalyanak Ghrit* is useful treat various diseases like *Jvara (fever), Kasa (cough), Shwas (respiratory-related disease), Unmad (psychiatric disorder), and Vishavikar* (poison or toxicity). It can target multiple pathologies at the same time, and this can improve various neuropsychiatric domains in post-COVID status [26].
- All of the above intervention drugs are useful in combating post-COVID-19-related symptoms, the top combination of drugs which are mentioned in *Jvara Chikitsa* (chapter of fever). It also acts on the respiratory system and improve the functional status similar to the symptoms of post-COVID-19 syndrome.

After COVID-19, viral infection leads to an aggressive immunological reaction on various systems and various diseases associated with an immune system and metabolism, and *Basti* therapy (medicated enema) in Ayurveda can modulate immune responses by regulating pro-inflammatory cytokines, immune globulins, and functional properties of T cells which were mentioned by Thatte et al. So, we have decided to see the effect of *Basti* and *Rasayana* on symptoms of post-COVID-19 syndrome.

Recently, a new variant B.1.1.529, named Omicron, has emerged, and their signs and symptoms as the same as SARS-COV-2 variant and other newer variants may emerge during the study research and see the modulation of immunogenicity and the action of the newer variant in various systems in the human body. It would be interesting to evaluate the responses by variant when taking interventions [27].

Our trial has the potential to have a significant impact on those taking excessive nutritious and poor nutritious diet after COVID-19 infection; this also serves as a protection to the general wellbeing of the patients with a safe, simple, adorable intervention in improving their immune responses through *Basti* and *Rasayana* therapy [13].

### Strength and limitations of this study

This is a proof of concept open-labeled pragmatic clinical study. In this study, the patients of post-COVID-19 syndrome will be treated on the basis of classical Ayurvedic concepts and formulations. The exact dose will also be estimated on the basis of the classical Ayurveda examination. This study will be helpful to understand the impact of the fundamentals of Ayurveda in clinical decision making and deciding the treatment.

This study is a part of a post-graduate thesis and is non-funded; hence, we are taking clinical scales only. Higher immunological and metabolic parameters are not tested in this study.

## Trial care and record retention

The participants will be advised to give a regular follow-up and inform about adverse events of intervention (if any) drug which will be given to the participants. All clinical study documents will be retained by the investigator for at least 5 years after the study. All protocol amendment will be approved by the respective Ethics Committees before implementation. In the event of any types of medical emergency or otherwise, the site investigator will inform the appropriate Ethics Committee, study coordinators within 24 h. The trial will be conducted in compliance with the protocol. Deviations from the protocol will not be made except when it is necessary to alleviate an immediate hazard to the trial patients. All the protocol amendments, including the changes to interventions, examinations, data collections, and methods of analysis, will be reported to IEC at the earliest along with the exact reason. Study-related information will be stored in the study site. All patient information will be stored securely in a lock system. All the data collected in case record forms will be stored. All records that contain names or other personal identifiers, such as informed consent forms, will be stored separately, and the study records will be identified by code numbers. The data will be identified by a coded number only to maintain the participant’s confidentiality.

## Supplementary Information

**Additional file 1.**

## Data Availability

After informing the patients about the research study, written consent and patient information sheet will be taken from the patients before the screening. After screening the patients, the eligible and willing patients will be included in the study and the data will be collected in a case record format. The data will be recorded in an e-format for the statistical analysis and storage of the record. The data analysis will be done by INSTAT 3 and SPSS. The data will be coded and entered in a MS-Excel work sheet, and then, this statistical software will be used. Data will be published in reputed journals after the completion of the study.

## Abbreviations

OPD: Outdoor patient department
IPD: Indoor patient department
BD: Twice a day
BMI: Body mass index
CRF: Case record form
