# Supplementary material for: The clinical evaluation of Basti along with Rasayana on symptoms of post-COVID-19 syndrome: an open-labeled proof of concept pragmatic study—a study protocol
Source: Pilot Feasibility Stud. 2023 Jun 3;9:92. doi: 10.1186/s40814-023-01322-1 (PMC10238229; doi:10.1186/s40814-023-01322-1)
Supplement: Supplementary file 1 — Additional file 1. [file 40814_2023_1322_MOESM1_ESM.docx]

| **Title-‘Clinical evaluation of *Basti along* with *Rasayana* on symptoms of Post COVID 19 Syndrome. Open labelled proof of concept pragmatic study.’**    **Patient complaints sheet-**   \| Name of Patient :…………………………….  ……………………………………………….. \| \| Address: \| \| --- \| --- \| --- \| \| Age :………Years \| \| \| Sex : Male/Female \| \| \| Registration No. OPD:  IPD: \| \| Religion : \| \| Date of Admission:…..../…….……../………. \| \| \| \| Educational Status: \| \| Profession: \| \| Social and Economic Status : VP/P/LM/M/UM/R/VR \| \| \| \| Diagnosis:………………………………………. \| Duration:……………………. Since………….. \| \| |
| --- | --- | --- | --- | --- | --- | --- | --- | --- | --- | --- | --- | --- | --- | --- | --- | --- | --- | --- | --- | --- | --- | --- |

**Case Record Form**

| **Chief complaints-**  **Table -17**   \| **S .NO** \| **Chief complaints** \| **Duration** \| **Gradation** \| \| --- \| --- \| --- \| --- \| \| **1** \| Chronic fatigue \|  \|  \| \| **2** \| Dyspnea \|  \|  \| \| **3** \| Shortness of breath \|  \|  \| \| **4** \| Chest pain \|  \|  \| \| **5** \| Headache \|  \|  \| \| **6** \| Loss of smell /taste \|  \|  \| \| **7** \| Muscle and joint pain \|  \|  \| \| **8** \| Depression \|  \|  \| \| **9** \| Anxiety \|  \|  \| \| **10** \| Insomnia \|  \|  \| \| **11** \| Itchy body \|  \|  \| \| **12** \| Heart palpitations \|  \|  \| \| **13** \| Tachycardia \|  \|  \| \| **14** \| Anorexia \|  \|  \| \| **15** \| Tingling fingertips \|  \|  \| \| **16** \| Brain fog \|  \|  \|   **Present complaint-**  **Past history of disease -**  **Medicinal history -**  **Family history- Mother-**  **Father-**  **Husband-**  **Wife-**  **Other-**    **Food Intake**:  **Dominant Taste**: taste of sweet/ sour/salty/pungent/bitter/Astringent    **Property, quality or attribute***:* Heaviness/Lightness/ Coldness/ hotness/Unctuousness, oiliness/Dryness/other…..  **Dietetic Habit:**  **Type of food**: Veg/Non-Veg  **Occupation :**  Working time: Day/Night/Shift  Nature of work: Manual/Sedentary/Laborious   1. **Addiction** : Alcohol/ Smoking/ tobacco/Betel nut /tea 2. **Hunger** : low/mid/high 3. **Thirst** : low/mid/high 4. **Sleep** : less/mid/more 5. **Relaxation**: 6. **Married life :** 7. **Peristalsis movement-** 8. **Menstrual history** *:*   *:*  ***Dash vidha Pariksha****: (*Ten domains of clinical examination)   1. ***Prakriti*** (An individual’s inherent nature)   ***Doshaj Prakriti*** (Body constitution with a predominance of dosha) : V/P/K/VP/PK/VK/VPK  ***Manas Prakriti*** (Specific mental and physical personality type) :  Satvik (Person with tranquil, harmonious temperament)  Rajasik (Person with agitated, reactive temperament)  Tamasik (Person with inertial temperament)  RT /SR/ST  **Built:** Krisha (lean)/Madhyam (normal)/Sthula(obese)  **Skin :** Dry/oily/normal  **Hair** : less /normal/oily/dry  **Strength** : poor /normal /good  **Behavior**: Anger/silent/Normal  *2.****Saratah*** *(*Predominance of a particular fundamental structural component in a person)*:*  *Twak(*Person with predominance or essence of primary circulating nutrient fluid)  *Rakta* (essence of blood)*/ Mansa* (essence of muscle)  *Meda* (essence of fat)  *Asthi* (essence of bone)  *Majja* (essence of bone marrow)  *Shukra* (essence of semen).  *3.****Samhanantah*** *(*body constitution) *:poor/normal/good*  4. **Pramantah** (Body Mass Index) : SharirAayam (Height):SharirBhar(Weight):  *5.****Satmyatah*** *(habits) :*  *6.****Satvatah*** *( body substance) :*  *7.****Aaharashakti*** *(*Food intake) : AbhyavaharanShakti: (food intake capacity)  JaranShakti: (digestive power)  8.**VyayamShakti** (capability to carry on physical activities):  9.**Age** : children / adult / old  10.Site : body site/ disease site  ***Ashtavidha Evam Samanya Parikshan:* (eight methods of clinical examination)**   1. Pulse : Regular/irregular- 2. Stool 3. Urine 4. Tongue : 5. Speech: 6. Temperature: Dehoshma 7. Vision 8. Body Built   ***Samanya Pariksha****:* (General Examination)   1. *Pulse :* 2. *Weight :* 3. *Temperature :* 4. *Blood pressure:* 5. *Nails :* 6. *Eyes :* 7. *Tongue :*   *8.Stool and urine :*  ***Strotas Parikshan****: (***examination of body channels conduit)**   1. ***Pranavaha Strotas****:* Conduit for vital life, respiratory tract.    1. Inspection:   Movement of Chest:  Rhythm:  Depth:  Expansion:   - 1. Palpation:   Position of trachea:  Tactile vocal fremitus:  Expansion: ……cm during inspiration  Movement of chest:   - 1. Percussion: Character of sound & Zone of Occurrence:   2. Auscultation: Breath sound:  1. Vesicular breathing: Normal/Increased/Decreased 2. Bronchial breathing: Tubular/Bronchial/Cavernous 3. Broncho vesicular breathing: Absent/Present 4. Adventitious sound:   i)Rhonchi: Absent/Present  ii)Crepitations: Absent/Present  Vocal Resonance: Normal/Increased/Decreased   1. ***UdakvahaSrotas***: Channel, pathway for fluid circulation  \| Symptom \| Jivha (tongue) \| Talu  (palate) \| Kantha  (Anterior portion of the neck) \| Oshtha  (lips) \| Kloma \| \| --- \| --- \| --- \| --- \| --- \| --- \| \| Dehydration \|  \|  \|  \|  \|  \|   Thirst:  Signs of dehydration:  Skin : Elasticity Dryness   1. ***AnnavahaSrotas***: Digestive tract   Oshtha (lips) - Jivha (tongue) -  Mukha (mouth) - Koshtha (bowe) –  Dant (teeth) - Grahani (abdomen) –   1. ***Rasavaha Srotas:*** Channel carrying nutrient fluids   Pulse- Blood pressure- Temperature-  Hridaya parikshan  a) Inspection: Venous pulsation in neck -  Arterial pulsation  b) Palpation: Apex beat -  Position of Apex beat -  Rhythm - R / IR / RIR / IRIR   1. Percussion:   Area of cardiac dullness - Usual/ diminished /  Increased  Rt / Lt / Upper Margin  d) Auscultation: Heart sound- Present  Intensity- Audible  Splitting - 1^st^Sound – Absent / Present  2^nd^ Sound – Absent / Present  Additional sound -Absent/Present  ***-***  ***5) Raktavaha Srotas:*** (Channel carrying blood)  Liver- P/N  Spleen- P/N  Artreies and veins-  Hb % -…………., TLC-……….., ESR-………..,  DLC -…………,  ***6) Mansavaha Srotas:*** (Conduit nourishing muscle)  Movement –  ***7) MedovahaSrotas:*** (Channel carrying fat)  Kidney – Inspection-  Palpation –  Abdomen –  Lumbar –  Hip-  Skin pores –  Sweat-  Weight –  ***8) AsthivahaSrotas:*** (Channel carrying bone)  Sandhi Parikshana ( joint examination)  (a). Darshana (inspection):  (b). Sparshan (Palpation)  (a). Prashna (Questionnaire):  ***9) MajjavahaSrotas:*** (Channel carrying bone marrow)  (small and large joint examination)    ***10) ShukravahaSrotas:*** (Channel carrying structural components of reproduction)  Scrotum including testicle -  Penis -  Semen-  **11)** **Artavavaha Srotas:** (female reproductive system)  Vagina –  Vaginal opening -  Uterus –  Menstruation -  ***12) PurishvahaSrotas:*** (Channel in which faeces is formed and excreted)  Intestine-  Anal-  feces –  ***12) Mutravaha Srotas:*** (urinary tract)  Ureter–  Urinary bladder –  genitals–  urine examination- –  Quantity -  , Odour (Gandha)-  Colour&Transperancy –  ***13) SvedavahaSrotas:*** (channel of sweat gland)  Sweating–  Pores –  ***Vriddi/Kshaya Lakshan:***  ***SrotodushtiLakshan-***  ***Vikrititaha Parikshan***   1. ***Dosha***: *Vata*- *Vata*, dosha that provides the attribute of life to an organism life force(*Pran*)/ Ascending *vata* (*Udan*) / Circulating *vata* (*Vyan*)/ Kindling *vata* (*Saman*)/descending *vata* (*Apan*)   *Pitta*-Digesting *pitta* (*Pachak)*/coloring *pitta* (*Ranjak*)/*pitta* for vision (*Alochaka*)/ *pitta* for complexion (*Bhrajak*)/*pitta* for intellect (*Sadhak*)  *Kapha*-supporting cough (*Avalambaka*)/moistening cough (*Kledaka*)/taste perceiving *kapha* (*Bodhaka*) /nourishing cough (*Tarpak*)/lubricating cough (*Shleshmaka*)   1. ***Dushya***: (A bodily structure which can be vitiated by aggravated doṣa)-   *Rasa* (Primary circulating nutrient fluid, primary product of digestion), *Rakta* (blood), *Mansa* (muscle), *Meda* (fat) , *Asthi* (bone), *Majja*, (bone marrow), *Shukra* (semen), *Purish* (stool), *Mutra* (urine), *Sveda* (sweat)   1. ***Srotovikrit*i: (Vitiation of channels)-** *Pranvaha* (Vitiation of channels carrying vital life force) , *Annavaha* (Vitiation of channels carrying food), *Udakvaha* (Vitiation of channels carrying water) , *Rasavaha*, (Vitiation of channels carrying primary product of digestion), *Raktavaha* (Vitiation of channels carrying blood) ,*Mansavaha* (Vitiation of channels carrying muscle), *Medovaha* (Vitiation of channels carrying medas), *Asthivaha* (Vitiation of channels carrying bone),*Majjavaha* (Vitiation of channels carrying bone marrow), Shukra (Vitiation of channels carrying semen), *Aartavaha* (Vitiation of channels carrying menstrual flow), *Purishvaha* (Vitiation of channels carrying stools), *Mutravaha* (Vitiation of channels carrying urine), *Svedavaha* (Vitiation of channels carrying sweat).   4) **Place**  5) **side effect**  **6) curable/incurable**  **7) condition**   1. **Relief-** 2. **Not Relief*:***   ***10)*Differential Diagnosis**  **Follow Up Observational Table-1**   \| **Sr.no.** \| **Signs and symptoms** \| **B.T.(zeroday)** \| **M.T. (after13^th^day)** \| **A.T. (after 35^th^day)** \| \| --- \| --- \| --- \| --- \| --- \| \| 1 \| Chronic fatigue \|  \|  \|  \| \| 2 \| Dyspnea \|  \|  \|  \| \| 3 \| Shortness of breath \|  \|  \|  \| \| 4 \| Chest pain \|  \|  \|  \| \| 5 \| Headache \|  \|  \|  \| \| 6 \| Loss of smell/taste \|  \|  \|  \| \| 7 \| Muscle and joint pain \|  \|  \|  \| \| 8 \| Depression \|  \|  \|  \| \| 9 \| Anxiety \|  \|  \|  \| \| 10 \| Insomnia \|  \|  \|  \| \| 11 \| Itchy body \|  \|  \|  \| \| 12 \| Heart palpitations \|  \|  \|  \| \| 13 \| Tachycardia \|  \|  \|  \| \| 14 \| Anorexia \|  \|  \|  \| \| 15 \| Tingling fingertips \|  \|  \|  \| \| 16 \| Brain fog \|  \|  \|  \|     **Follow up observational table-2**     \| **Examination** \| **Day 0** \| **Day 5** \| **Day 6-14** \| **Day 35** \| \| --- \| --- \| --- \| --- \| --- \| \| Mala (stool) \|  \|  \|  \|  \| \| Mutra(urine) \|  \|  \|  \|  \| \| Shuddha (hunger) \|  \|  \|  \|  \| \| Nidra (sleep) \|  \|  \|  \|  \| \| Pipasa (thirst) \|  \|  \|  \|  \| \| BP \|  \|  \|  \|  \| \| PR \|  \|  \|  \|  \| \| SPO2 \|  \|  \|  \|  \|     **Follow up Observation Table -3**     \| **Scales** \| **Day 0** \| **Day 14** \| **Day 35** \| \| --- \| --- \| --- \| --- \| \| Fatigue severity scale \|  \|  \|  \| \| MMRC Dyspnea scale \|  \|  \|  \| \| Chest pain scale \|  \|  \|  \| \| VAS scale \|  \|  \|  \| \| Smell and Taste scale \|  \|  \|  \| \| WOMAC scale \|  \|  \|  \| \| Hamilton depression scale \|  \|  \|  \| \| Hamilton anxiety scale \|  \|  \|  \| \| Insomnia severity index \|  \|  \|  \| \| Cough severity index \|  \|  \|  \| \| Facial aging scale \|  \|  \|  \| \| Dizziness scale \|  \|  \|  \| \| Pittsburge severity quality index \|  \|  \|  \| \| Functional status scale \|  \|  \|  \| \| Heart palpitation scale \|  \|  \|  \|   **Follow up Observation table -4**   \| Sr.  No. \| Physical Character \| B.T. (zeroday) \| A.T(After 35 days) \| \| --- \| --- \| --- \| --- \| \| 1 \| Height \|  \|  \| \| 2. \| Weight \|  \|  \| \| 3. \| BMI \|  \|  \| \| 4. \| Abdominal circumference \|  \|  \| \| 5. \| Waist circumference \|  \|  \| \| 6. \| Hip circumference \|  \|  \| \| 7. \| Skin fold-shoulder \|  \|  \| \| 8. \| Skin fold-abdomen \|  \|  \|     **Follow Up Observational Table-5**   \| **S.N** \| ***Basti*  type (therapeutic enema)** \| ***Basti dana kal* (time of administration)** \| ***Basti Pratyagaman* ( return time of enema)** \| **M*atra***  **(dose)** \| ***Lakshan* (Symptoms)** \| \| --- \| --- \| --- \| --- \| --- \| --- \| \| **1** \|  \|  \|  \|  \|  \| \| **2** \|  \|  \|  \|  \|  \| \| **3** \|  \|  \|  \|  \|  \| \| **4** \|  \|  \|  \|  \|  \| \| **5** \|  \|  \|  \|  \|  \| \| **6** \|  \|  \|  \|  \|  \| \| **7** \|  \|  \|  \|  \|  \| \| **8** \|  \|  \|  \|  \|  \| |
| --- | --- | --- | --- | --- | --- | --- | --- | --- | --- | --- | --- | --- | --- | --- | --- | --- | --- | --- | --- | --- | --- | --- | --- | --- | --- | --- | --- | --- | --- | --- | --- | --- | --- | --- | --- | --- | --- | --- | --- | --- | --- | --- | --- | --- | --- | --- | --- | --- | --- | --- | --- | --- | --- | --- | --- | --- | --- | --- | --- | --- | --- | --- | --- | --- | --- | --- | --- | --- | --- | --- | --- | --- | --- | --- | --- | --- | --- | --- | --- | --- | --- | --- | --- | --- | --- | --- | --- | --- | --- | --- | --- | --- | --- | --- | --- | --- | --- | --- | --- | --- | --- | --- | --- | --- | --- | --- | --- | --- | --- | --- | --- | --- | --- | --- | --- | --- | --- | --- | --- | --- | --- | --- | --- | --- | --- | --- | --- | --- | --- | --- | --- | --- | --- | --- | --- | --- | --- | --- | --- | --- | --- | --- | --- | --- | --- | --- | --- | --- | --- | --- | --- | --- | --- | --- | --- | --- | --- | --- | --- | --- | --- | --- | --- | --- | --- | --- | --- | --- | --- | --- | --- | --- | --- | --- | --- | --- | --- | --- | --- | --- | --- | --- | --- | --- | --- | --- | --- | --- | --- | --- | --- | --- | --- | --- | --- | --- | --- | --- | --- | --- | --- | --- | --- | --- | --- | --- | --- | --- | --- | --- | --- | --- | --- | --- | --- | --- | --- | --- | --- | --- | --- | --- | --- | --- | --- | --- | --- | --- | --- | --- | --- | --- | --- | --- | --- | --- | --- | --- | --- | --- | --- | --- | --- | --- | --- | --- | --- | --- | --- | --- | --- | --- | --- | --- | --- | --- | --- | --- | --- | --- | --- | --- | --- | --- | --- | --- | --- | --- | --- | --- | --- | --- | --- | --- | --- | --- | --- | --- | --- | --- | --- | --- | --- | --- | --- | --- | --- | --- | --- | --- | --- | --- | --- | --- | --- | --- | --- | --- | --- | --- | --- | --- | --- | --- | --- | --- | --- | --- | --- | --- | --- | --- | --- | --- | --- | --- | --- | --- | --- | --- | --- | --- | --- | --- | --- | --- | --- | --- | --- | --- | --- | --- | --- | --- | --- | --- | --- | --- | --- | --- | --- | --- | --- | --- | --- | --- | --- | --- | --- | --- | --- | --- | --- | --- | --- | --- | --- | --- | --- | --- | --- | --- | --- | --- |
